# Exploring the antidiabetic potential of *Octhochloa compressa*: a comprehensive study on chemical profiling, *in vitro*, *in vivo* and *in silico* pharmacological properties

**DOI:** 10.3389/fphar.2025.1541482

**Published:** 2025-05-20

**Authors:** Jawaria Aslam, Mirza Imran Shahzad, Hanan Y. Aati, Kashif-ur-Rehman Khan, Maria Aslam, Athar Jamal, Umar Farooq, Bilal Ahmad Ghalloo

**Affiliations:** ^1^ Department of Physiology and Biochemistry, Cholistan University of Veterinary and Animal Sciences (CUVAS), Bahawalpur, Pakistan; ^2^ Department of Biochemistry, Institute of Biochemistry, Biotechnology and Bioinformatics, The Islamia University of Bahawalpur, Bahawalpur, Pakistan; ^3^ Department of Pharmacognosy, College of Pharmacy, King Saud University, Riyadh, Saudi Arabia; ^4^ Depertment of Pharmaceutical Chemistry, Faculty of Pharmacy, The Islamia University of Bahawalpur, Bahawalpur, Pakistan; ^5^ Department of Information Technology, The Islamia University of Bahawalpur, Bahawalpur, Pakistan; ^6^ Department of Chemistry, The University of Lahore, Lahore, Pakistan; ^7^ Department of Computer Science, Cholistan University of Veterinary and Animal Sciences (CUVAS), Bahawalpur, Pakistan; ^8^ Department of Medicinal Chemistry, College of Pharmacy, University of Minnesota, Minneapolis, MN, United States

**Keywords:** *in vitro* anti-diabetic, *in silico* anti-diabetic, *in vivo* anti-diabetic, *Octhochloa compressa*, alloxan

## Abstract

Diabetes can lead to various health complications but can be managed with medication, diet, lifestyle changes, and certain medicinal plants with antidiabetic properties. *Octhochloa compressa,* a plant native to arid regions with a history of medicinal use, is being comprehensively examined for the first time using *in vitro, in silico*, and *in vivo* approaches to evaluate its efficacy in combating diabetes. *In vitro* α-glucosidase inhibition assays were performed using aqueous, methanol, *n*-butanol, ethyl acetate, *n*-hexane, and dichloromethane extracts. The ethyl acetate (EtAc) and methanol (MetOH) extracts showed the strongest inhibition with IC_50_ values of 190.6 ± 1.19 μg/mL and 281.0 ± 0.98 μg/mL, respectively. *In vivo*, the anti-diabetic activity of aqueous, MetOH, and EtAc extracts was assessed at 250, 500, and 750 mg/kg body weight in alloxan-induced hyperglycemic rabbits (blood glucose >250 mg/dL). A 30-day study revealed that EtAc extract at 500 mg/kg significantly reduced glucose levels from 328.38 ± 0.86 mg/dL to 121.61 ± 1.28 mg/dL (P < 0.001), along with notable improvements in serum bilirubin, lipid profile, creatinine, ALT, and AST levels compared to the negative control. Histopathological analysis showed no toxic effects on liver, kidney, or adrenal tissues. HPLC analysis of the potent EtAc extract identified bioactive compounds, and *in silico* docking revealed that tannins, gallic acid, coumarin, oxindole, and xanthone formed stable complexes with α-glucosidase (PDB ID: 3W37), with docking scores of −7.91, −6.59, −6.34, −6.33, and −6.07 kcal/mol, respectively. These findings suggest that *O. compressa* contains active compounds with significant anti-diabetic properties and minimal toxicity, making it a promising candidate for diabetes management and its complications. Future research should isolate and characterize key bioactive compounds and validate their mechanisms, safety, and clinical efficacy to advance *O. compressa* as a potential antidiabetic therapy.

## 1 Introduction

Diabetes mellitus (DM) is a chronic metabolic disorder resulting from abnormal insulin production or metabolism, leading to chronic hyperglycemia, characterized by disturbances in carbohydrate, lipid, and protein metabolism, along with symptoms like polydipsia, polyuria, polyphagia, high urine glucose, and weight loss (Hilal et al., 2023; Maqbool et al., 2021). Approximately 7% of adults worldwide suffer from DM, with a sharp increase paralleling obesity rate. The International Diabetes Federation predicts that by 2030, there will be approximately 552 million diabetic patients. There are two major types of DM: Type 1 (T1DM) and Type 2 (T2DM), with T2DM being more prevalent (95%–90% of cases) (Martínez-Venegas et al., 2019; Su et al., 2022). Currently, clinical treatments for DM include insulin and oral anti-diabetic agents, like glucosidase inhibitors, biguanides, insulin sensitizers, and sulfonylureas. However, many of these medications have limitations and side effects, such as liver and kidney issues, hypoglycemia, diarrhea, and lactic acidosis (Nurdianti et al., 2020). To address these concerns and find more effective and safer alternatives, researchers are increasingly exploring natural compounds derived from traditional herbal medicines. These traditional remedies, endorsed by the World Health Organization (WHO), hold promise as valuable resources for developing new therapeutic anti-diabetic drugs for DM patients (Khan et al., 2022; Patil et al., 2020).

Medicinal plants are highly valued for their potential in discovering therapeutic compounds and as a resource for new drug development. The World Health Organization (WHO) supports their use in both modern and traditional medicine (Aslam et al., 2023a; Jugran et al., 2021). The pharmaceutical industry increasingly relies on natural sources for medications. Natural compounds from medicinal plants, with their diverse pharmacological activities and structural variety, offer unique advantages in identifying novel lead compounds for various targets, including antioxidant, anticancer, antimicrobial, and enzyme inhibition properties. Natural products continue to inspire breakthroughs in chemistry, biology, and medicine (Elangovan et al., 2019). Numerous medicinal plants such as *Momordica charantia* (charantin) (Parra et al., 2021), Withania frutescens L. (Mechchate et al., 2021), and Coronopus didymus (Muzammil et al., 2022), have demonstrated significant antidiabetic activity through mechanisms such as enhancing insulin secretion, improving insulin sensitivity, and inhibiting carbohydrate-digesting enzymes*.* These bioactive constituents exhibit promising glucose-lowering effects and are being further investigated for potential development into standardized herbal formulations or lead compounds for antidiabetic drugs. Thus, medicinal plants are indispensable in the search for new therapeutic agents, particularly in addressing the global burden of diabetes, and continue to play a critical role in advancing medical science (Nazir et al., 2021).

*Octhochloa compressa*, also known as Chimbar, Hillu, Phalwan, or Ghora dhob, is a perennial plant in the *Poaceae* family, with a growth period from April to September. It features narrow, pointed leaves with a hairy base, measuring 5 cm in length and 2 mm in width, and its spikes, approximately 1.5–1.4 cm long, contain 2-3 florets (Shameem Kausar and Hameed, 2023). *O. compressa* has a wide geographical distribution across Africa, Arabia, Pakistan, and India, contributing significantly to diverse ecosystems. It has been observed in regions including Kohat, D.I. Khan, Rawalpindi, Mardan, Karachi, Thar Parker, Hyderabad (especially near Narwari and Soon Sakesar), Kariala, Chakwal, and Dhok Seela (Al-Sodany et al., 2011). While its presence may be rarer in some areas, it tends to thrive near mountains, slopes, and even wildlife sanctuaries in Chakwal, where it adapts well to clay soil. This adaptability and its role as a valuable fodder resource make *O. compressa* essential for sustaining both livestock and wildlife across these diverse landscapes (Ahmad et al., 2011; Chaudhari et al., 2014). Despite its ecological significance, *O. compressa* remains largely unexplored pharmacologically. The only known scientific report to date describes its *in vitro* and *in vivo* anti-inflammatory potential, as demonstrated in our recent study (Aslam et al., 2021). Although there are currently no documented ethnomedicinal reports directly linking *O. compressa* to diabetes management, anecdotal uses among nomadic communities in southern Punjab suggest a potential role in managing metabolic imbalances. Moreover, several members of the Poaceae family are known to contain phytochemicals with antidiabetic properties, including flavonoids, alkaloids, and polyphenols, which supports the rationale for exploring *O. compressa* in this context (Hussein et al., 2018). Therefore, in our ongoing efforts to identify new natural compounds with therapeutic relevance, this study aims to evaluate the *in vitro, in silico,* and *in vivo* antidiabetic effects of *O. compressa* extracts.

## 2 Materials and methods

### 2.1 Plant collection and extraction

**FIGURE 1:**
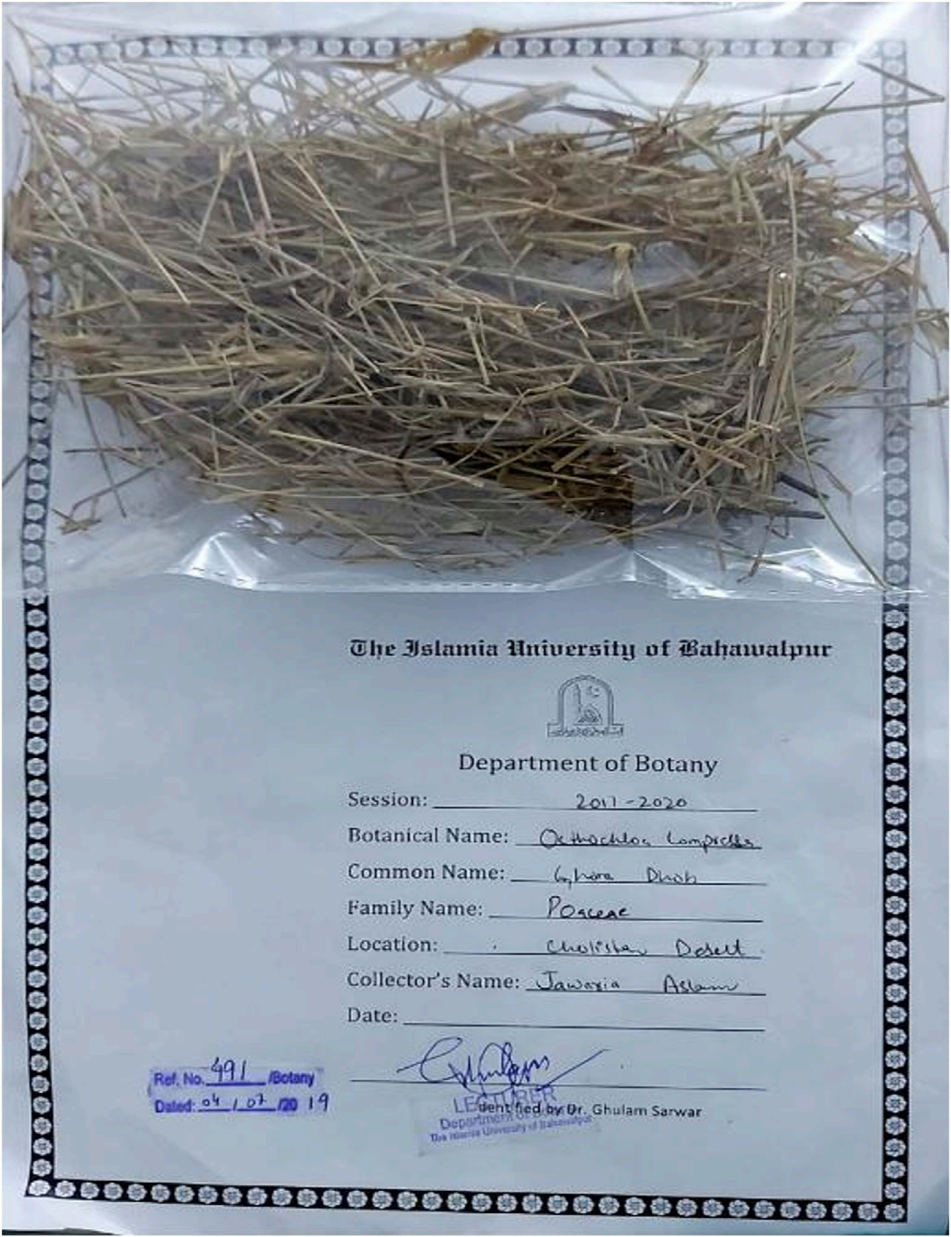
Identification and voucher number of *O. compressa*.

The entire *Octhochloa compressa* plant was collected from the Cholistan desert, situated near Bahawalpur city in Punjab, Pakistan. The plant’s authenticity was confirmed by a taxonomist from the Botany Department at The Islamia University of Bahawalpur, evidenced by voucher number Oc-491 and date 04-02-2019 (Figure 1). For extraction, the whole plant underwent washing, followed by shade drying and subsequent grinding into a fine powder. The dried plant powder of 250 g was immersed in 2000 mL of six distinct solvents: *n-*hexane, dichloromethane, *n-*butanol, ethyl acetate, methanol, and aqueous solution. This mixture was allowed to soak for 72 h, with intermittent shaking, each mixture was subjected to filtration, and the resulting extracts were concentrated using a rotary evaporator. The yield of each extract was then calculated based on the initial plant material (Aslam et al., 2023b).

### 2.2 *In vitro* anti-diabetic assay

The α-glucosidase inhibition assay, based on recent method with slight modifications (Huang et al., 2019), involved the addition of phosphate buffer (50 µL), α-glucosidase (10 µL), and pre-prepared extracts (20 µL) to the wells, followed by a 15-min incubation at 37°C. All extracts were prepared as 10 mg/mL stock solutions in methanol solvent; sticky semi-dried extracts underwent further drying for accurate weighing. The final concentration of extract in the reaction mixture was calculated based on the stock solution concentration. After the incubation, P-NPG (20 µL) was introduced as a substrate and incubated for an additional 20 min at 37°C. The reaction was halted by adding Na_2_CO_3_ (50 µL), and absorbance was recorded at 405 nm. This assay, performed in triplicate and IC_50_ values was calculated by GraphPad Prism.

$$\%\;inhibition=\frac{Ac-At}{Ac\;}\times 100$$

Where, Ac is the absorbance of control and At is the absorbance of test sample.

### 2.3 *In vivo* anti-diabetic assay

#### 2.3.1 Experimental animals

Rabbits of both sexes, weighing between 1.5 and 2.0 kg, were housed in Zone-2 of the animal facility within the research laboratory of the Department of Pharmacy, Faculty of Pharmacy and Alternative Medicine at The Islamia University of Bahawalpur. The rabbits were maintained under standard laboratory conditions, which included a 12-h light/dark cycle, a temperature of 22°C ± 2°C, and humidity levels between 35% and 60%, with unrestricted access to a standard diet and water (Ibrahim et al., 2021). Rabbits were selected based on their proven sensitivity to alloxan-induced β-cell cytotoxicity, their suitability for long-term antidiabetic studies, and their ability to tolerate repeated blood sampling—an essential feature for monitoring glycemic responses over time—making them a reliable and appropriate model for evaluating the hypoglycemic effects of plant-based extracts (Maqbool et al., 2021).

#### 2.3.2 Experimental design

After a 1-week adaptation period, a group of rabbits were fasted overnight for 8 h. Among them, five rabbits were randomly selected as the normal base line group (NG), which received no interventions. The remaining rabbits were intravenously injected with alloxan monohydrate at a dose of 120 mg/kg body weight to induce hyperglycemia. Blood glucose levels were monitored from the ear vein using a glucometer (Accusign MM 1000) in the overnight fasted rabbits on the third to fifth day after alloxan injection. Selected hyperglycemic rabbits (>250 mg/dL FBGL) were randomly divided into 12 groups (each group with n = 5 animals), included a negative control (treated with normal saline), a positive control (treated with Glibenclamide), and various experimental groups that received different doses of *O. compressa* aqueous, methanol, and ethyl acetate extracts. In accordance with established methodologies, the current study did not include an extract-only control group, as the safety of *O. compressa* extracts at the administered doses (250, 500, and 750 mg/kg BW) has been previously validated in toxicity studies (Aslam et al., 2021). Based on prior evidence of safety and antidiabetic efficacy, *O. compressa* extract doses (250, 500, 750 mg/kg BW) were used for glucose-lowering assessments, consistent with dose ranges used for medicinal plants like *Cynodon dactylon*, *Cucumis trigonus*, and *Semecarpus anacardium* to evaluate dose-dependent effects (Ali et al., 2015). Blood glucose levels were measured and recorded on days 5, 8, 11, 14, 17, 21, and 30 of the study using a glucometer (Accusign MM 1000) (Elangovan et al., 2019).

**Table:** 

Group Ⅰ	Normal base line control (without any disease induction)
Group ⅠⅠ	Negative Control (Alloxan induced + Normal saline 5 mL/kg BW)
Group ⅠⅠⅠ	Positive Control (Alloxan induced + Glibenclamide 5 mg/kg BW)
Group Ⅳ	Alloxan induced + *O. compressa* Aqu extract of 250 mg/kg BW
Group Ⅴ	Alloxan induced + *O. compressa* Aqu extract of 500 mg/kg BW
Group Ⅵ	Alloxan induced + *O. compressa* Aqu extract of 750 mg/kg BW
Group Ⅶ	Alloxan induced + *O. compressa* MetOH extract of 250 mg/kg BW
Group Ⅷ	Alloxan induced + *O. compressa* MetOH extract of 500 mg/kg BW
Group ⅠⅩ	Alloxan induced + *O. compressa* MetOH extract of 750 mg/kg BW
Group Ⅹ	Alloxan induced + *O. compressa* EtAc extract of 250 mg/kg BW
Group Ⅺ	Alloxan induced + *O. compressa* EtAc extract of 500 mg/kg BW
Group Ⅻ	Alloxan induced + *O. compressa* EtAc extract of 750 mg/kg BW

#### 2.3.3 Biochemical parameters

The Blood samples of selective groups were collected, and serum was obtained by centrifuged at 2000 r/min for 12 min at room temperature. These samples were then used to assess various biochemical parameters, including total cholesterol, lipid profile, phospholipid levels, total bilirubin (T-BIL), total protein (TP), aspartate aminotransferase (AST), alkaline phosphatase (ALP), and alanine aminotransferase (ALT) using commercial kits. The analyses were conducted at the Pathology Laboratory within the Department of Pathology at Quaid-e-Azam Medical College in Bahawalpur, following the guidelines provided in the kit instructions (Elangovan et al., 2019).

#### 2.3.4 Histopathological analysis of tissues

Tissue samples from the kidney, liver, and adrenal gland were obtained and subsequently fixed in 10% buffered formalin on the last day of trial. The samples were then dehydrated using alcohol and embedded in paraffin. Thin paraffin sections were cut from these samples and subjected to hematoxylin-eosin staining. Histopathological alterations in the mentioned organs were observed at 100x magnification using an Olympus DP50 Digital camera system and documented through image recording with the assistance of image-pro Insight analysis software (Nazir et al., 2021).

### 2.4 HPLC polyphenol quantification

The HPLC-PDA (High-Performance Liquid Chromatography with Photodiode detector Array) quantification of 25 important polyphenolic compounds in EtAc extracts of the *O. compressa* was conducted using a standard protocol (Sahu et al., 2018).

#### 2.4.1 Sample preparation

HPLC–PDA (analysis was performed as follows: plant extracts were weighed on analytical balance and solubilized in mobile phase A (milliQ water +0.001 M acetic acid): B (acetonitrile +0.001 M acetic acid) (93:7, *v: v*), adding 20% dimethyl sulfoxide (DMSO), except for the aqueous plant extracts, which were perfectly soluble in only mobile phase. The samples were prepared at the concentration of 1 mg/250 µL. All samples were properly vortexed for ½ min, sonicated for 15 min and then were injected (20 µL) in HPLC system for the analysis. Control runs were also included to ensure accuracy and system performance, comprising a blank run (mobile phase only), standard run (known analyte solution), and a system suitability test (SST) to assess chromatographic parameters such as resolution and peak symmetry (Shen et al., 2021).

#### 2.4.2 HPLC conditions

The HPLC analysis was carried out on a Waters liquid chromatograph equipped with a model 600 solvent pump and 2,996 photodiode array detector (PDA). A C_18_ reversed–phase packing column (Prodigy ODS (3), 4.6 × 150 mm, 5 μm; Phenomenex, Torrance, CA, United States) was thermostated at 30°C ± 1°C using a Jetstream 2 Plus column oven. The UV/Vis acquisition wavelength was set in the range of 200–500 nm and the quantitative analyses were achieved at maximum wavelength for each compound. The injection volume was 20 μL and mobile phase was directly on–line degassed by using Biotech DEGAS, mod. Compact (Lab Service, Anzoladell, Emilia, Italy). Gradient elution was performed using the mobile phase water-acetonitrile (93:7, *v:v*, 3% acetic acid) and the concentration of each target compound was calculated using the calibration curve (Table 1).

**TABLE 1:** The gradient elution ratio of mobile phase in HPLC.

Time (min)	Flow (mL min^−1^)	%A	%B
0		93	7
0.1		93	7
30		72	28
38	1	75	25
45		2	98
47		2	98
48		93	7
58		93	7

### 2.5 *In silico* study of *O. compressa* extracts

#### 2.5.1 Acquisition of target protein and ligands

An *in silico* analysis was conducted for enzyme-inhibitor complexes utilizing the powerful Schrodinger software suite (https://www.schrodinger.com/). To commence, the three-dimensional structure of the target protein, α-Glucosidase (PDB ID: 3W37), was obtained from the Protein Data Bank (PDB, www.rcsb.org (Riyaphan et al., 2021)). For this study, the 2D molecular structures of the ligands were retrieved from PubChem (https://pubchem.ncbi.nlm.nih.gov), specifically Tannins (PubChem ID: 16129778), Gallic Acid (PubChem ID: 370), Coumarin (PubChem ID: 323), Oxindole (PubChem ID: 321710), Xanthone (PubChem ID: 7020), Apigenin (PubChem ID: 5280443), Quinoline (PubChem ID: 7047), Chromone (PubChem ID: 10286), Quinolizidine (PubChem ID: 119036), Acridone (PubChem ID: 2015), Caffeine (PubChem ID: 2519), Carboline (PubChem ID: 105078), Imidazole (PubChem ID: 795), and Lignan (PubChem ID: 261166).

#### 2.5.2 Preparation of protein and ligands for *in silico* study

To prepare this protein for precise docking simulations within the Maestro platform of Schrodinger, several critical steps were implemented. Initially, undesired amino acid residues and co-crystallized ligands were removed, the proton state of the protein was optimized using Epik, water orientations were sampled, and hydrogen bonds were assigned via Propka. The protein underwent further refinement through minimization, employing the Optimized Potential of Liquid Simulations (OPLS)-2005 force field, these 2D structures of test compounds were imported from the project table and subjected to modeling using the Ligprep module. All compound structures were geometrically optimized at their optimal pH, considering possible ionization and tautomeric states.

#### 2.5.3 Molecular docking

To facilitate precise docking, a receptor grid was generated tailored to the catalytic site of the α-glucosidase protein, guided by relevant literature (a grid box with grid dimensions were 16 × 24 × 28, and the coordinates for the center of the grid were set as x = 0.11, y = −2.511, and z = −22.097) (Azimi et al., 2021). These ligands were then flexibly docked to this grid using the Glide application, employing the standard precision (SP) mode with 32 runs per ligand and acarbose was used as a standard. Subsequently, a comprehensive qualitative and quantitative analysis of the Glide results was conducted, focusing on binding interactions and reporting docking scores as SP G Score in kcal/mol (Riyaphan et al., 2021; Tshiyoyo et al., 2022).

### 2.6 Statistical analysis

The study employed one-way ANOVA for statistical analysis, followed by Dunnet’s t-test with significance levels at p < 0.001, p < 0.01, and p < 0.05. GraphPad Prism 9 software was used, and results are presented as mean values with standard error of the mean (SEM) (Nazir et al., 2021).

## 3 Results

### 3.1 *In vitro* α-glucosidase inhibition by *O. compressa* extracts

*O. compressa* extracts were tested for α-glucosidase inhibition at different concentrations, revealing significant enzyme inhibition at higher concentrations. The highest α-glucosidase inhibition was observed by EtAc and MetOH extracts, with IC_50_ values of 190.6 ± 1.19 μg/mL and 281.0 ± 0.98 μg/mL, respectively, followed by Aqu extract with an IC_50_ of 380.5 ± 1.12 μg/mL *n-*But and DCM extracts also displayed considerable *α-*glucosidase inhibition at higher concentrations. The lowest activity was exhibited by *n*-Hex extract, with the highest IC_50_ value. The ranking of α-glucosidase inhibition activity for *O. compressa* extracts was EtAc > MetOH > Aqu > DCM > *n*-But > *n-*Hex (Table 2).

**TABLE 2:** Antidiabetic potential of *O. compressa* extracts by *α*-glucosidase inhibition.

Test samples	IC_50_ (μg/mL)
Acarbose	87.32 ± 0.19
Oc.Aqu	380.5 ± 1.12
Oc.MetOH	281.0 ± 0.98
Oc.*n*-But	452.4 ± 0.89
Oc.EtAc	190.6 ± 1.19^**^
Oc.*n*-Hex	688.1 ± 0.12
Oc.DCM	342.8 ± 2.17

The values are represented as Mean ± SEM, of triplicate in each group. The results are analyzed using two-way ANOVA., The comparisons are made with control group and results are considered significant (*) if P ˂ 0.05, more significant (**) if P ˂ 0.01 and highly significant (***) if P ˂ 0.001. Acarbose is used as a positive control. Oc.Aqu = aqueous, Oc.MetOH, methanol, Oc.*n*-But = *n*-butanol, Oc.EtAc = ethyl acetate, Oc.*n*-Hex = *n-*hexane, Oc.DCM, dichloromethane extracts of *Octhochloa compressa*.

### 3.2 *In vivo* anti-diabetic assay by *O. compressa* extracts in alloxan induced rabbits

**FIGURE 2:**
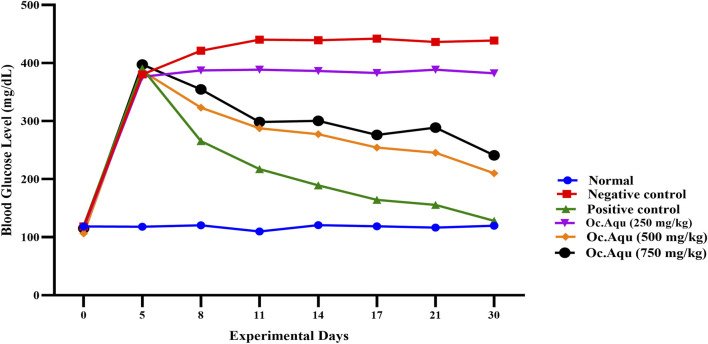
Anti-diabetic effect of *O. compressa* Aqu extract on diabetic induced rabbits.

**FIGURE 3:**
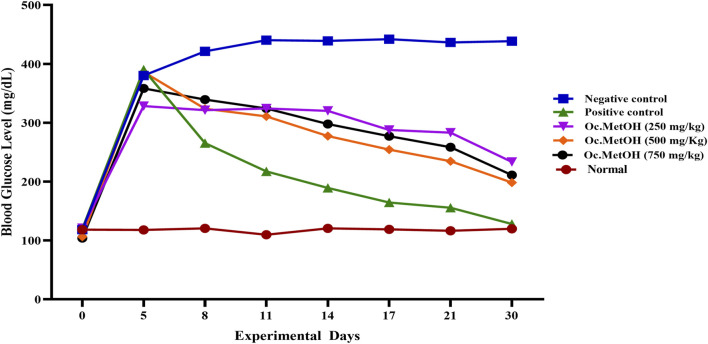
Anti-diabetic effect of *O. compressa* MetOH extract on diabetic induced rabbits.

**FIGURE 4:**
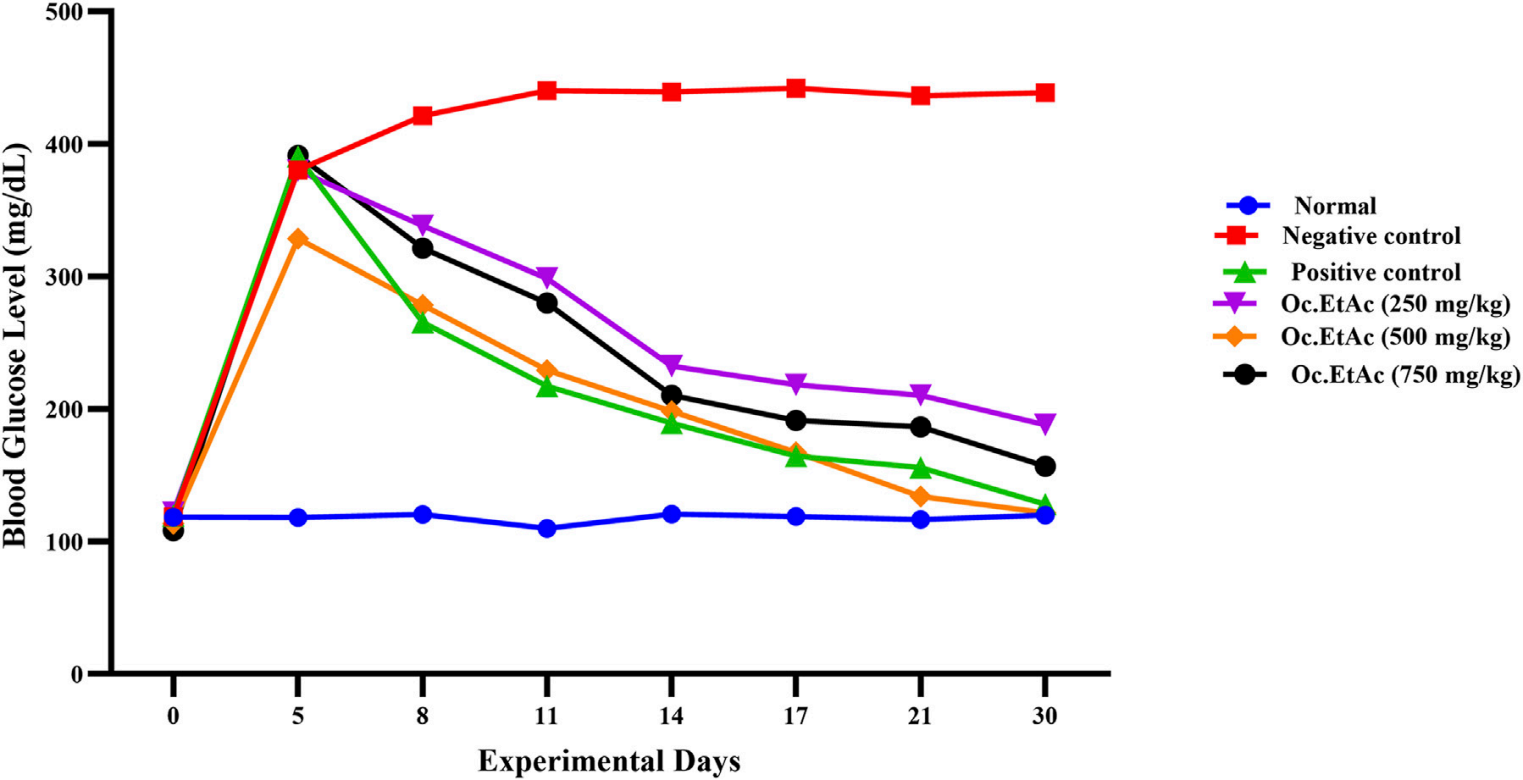
Anti-diabetic effect of *O. compressa* EtAc extract on alloxen diabetic induced rabbits.

The hypoglycemic effect of *O. compressa* crude extracts were evaluated on healthy and alloxan induced diabetic rabbits and results are summarized in Figures 2–4. Among 12 groups, group Ⅰ, normal base line group’s animal, having no intervention of disease, has shown no change in fasting blood glucose levels, in throughout the study. The group ⅠⅠ, negative control group, subjected to alloxan induction and treated with normal saline, exhibited a consistent increase in fasting blood glucose levels, signifying prolonged hyperglycemia. On the 21st and 30th days, fasting blood glucose levels reached 478.38 ± 1.12 mg/dL and 488.71 ± 0.85 mg/dL, respectively, indicating that normal saline did not influence blood glucose reduction. In the group ⅠⅠⅠ, standard group, glibenclamide treatment significantly lowered fasting blood glucose levels (P < 0.05) compared to the negative control. Subsequently, blood glucose dropped to 189.19 ± 0.87 mg/dL and 164.35 ± 1.0 mg/dL from an initial 390.37 ± 1.76 mg/dL on the 14th and 17th days, with highly significant (P < 0.001) effects. On the 21st and 30th days, fasting blood glucose levels were 155.68 ± 1.19 mg/dL and 127.98 ± 0.87 mg/dL respectively, confirming the standard medicine’s significant (P < 0.001) reduction of hyperglycemia in diabetic-induced rabbits.

In the group IV, V and VI, after alloxan induction, fasting blood glucose levels significantly increased, reaching 376.32 ± 1.65 mg/dL, 386.16 ± 2.52 mg/dL, and 397.33 ± 1.26 mg/dL for the respective dose groups on the 5th day of the experiment. The group ⅠⅤ, treated with 250 mg/kg BW Aqu extract of *O. compressa* showed no reduction in hyperglycemia and even led to a hyperglycemic condition. The 500 mg/kg BW Aqu extract of *O. compressa* group (group V) exhibited slight but insignificant reductions in hyperglycemia until the 17th day, with significant reductions observed on the 21st and 30th days, resulting in a FBGL of 210.12 ± 2.08 mg/dL on the 30th day. The group VI, treated with 750 mg/kg BW Aqu extract of *O. compressa* showed a slight reduction in hyperglycemia from 397.33 ± 1.26 mg/dL to 289.96 ± 1.33 mg/dL from the 3rd to the 14th day, with a significant (P < 0.05) difference compared to the control group. Overall, among all the doses of Aqu extract of *O. compressa*, only the 750 mg/kg BW dose exhibited a slight reduction in diabetes over the 30-day study period (Figure 2).

The group VII (treated with 250 mg/kg BW MetOH extract of *O. compressa*) exhibited a slight reduction in fasting blood glucose levels from 8th to 17th days, while significant (P < 0.01) hypoglycemic effect was observed on 30th day in diabetes-induced rabbits. The group VIII, treated with 500 mg/kg BW MetOH extract of *O. compressa* exhibited persistent reductions in hyperglycemia from the 8^th^ to the 17th day, reaching 254.26 ± 0.98 mg/dL and displaying significant (P < 0.01) hypoglycemia. Highly significant (P < 0.01) reductions in FBGL were observed on the 21st and 30th days. The 750 mg/kg BW MetOH extract of *O. compressa* group (group IX) also showed a slight reduction in hyperglycemia on the 8th and 11th days, with significant (P < 0.01) reductions on the 14th and 17th days. On the 21st and 30th days, highly significant (P < 0.01) reductions in FBGL were observed. Overall, the 500 mg/kg BW dose of MetOH extract of *O. compressa* was the most effective in maintaining blood glucose levels in diabetes-induced rabbits over the 30-day trial (Figure 3).

The groups X, treated by 250 mg/kg BW EtAc extract of *O. compressa*, significant (P < 0.01) reductions of hyperglycemia were observed from the 14th to the 21st day, while significant (P < 0.001) hypoglycemic effect were exhibited on the 30th day. In the 500 mg/kg BW EtAc extract of *O. compressa* group, fasting blood glucose levels reduced significantly (P < 0.05) from 328.38 ± 0.86 mg/dL before treatment to 278.51 ± 1.09 mg/dL on the 8^th^ day. The hypoglycemic effect continued to be highly significant from the 11th to the 17th day, with a highly significant (P < 0.01) reduction of FBGL to 121.61 ± 1.28 mg/dL on the 30th day. In the 750 mg/kg BW EtAc extract of *O. compressa* group, fasting blood glucose levels decreased insignificantly on the 5^th^ day, but significant and highly significant reductions in hyperglycemia were observed from the 11th to 30th day, the hyperglycemic reduction reached 156.73 ± 1.13 mg/dL (Figure 4). In the 30-day trial, among all selected extracts of *O. compressa*, the EtAc extract of *O. compressa* at the dose of 500 mg/kg BW significantly maintained blood glucose levels in hyperglycemic rabbits, indicating dose-dependent hypoglycemic effects.

### 3.3 Biochemical parameters

In this study, alloxan-induced diabetes in rabbits resulted in elevated bilirubin levels, which were significantly reduced by *O. compressa* extracts with antidiabetic potential, demonstrating anti-hyperbilirubinemia effects. The administration of these plant extracts did not lead to significant changes in serum creatinine levels, indicating no adverse effects on kidney function. Moreover, the extracts helped lower serum triglyceride levels, countering the increase observed in untreated diabetic rabbits, thus suggesting positive effects on metabolic status and lipid metabolism (p > 0.05). Overall, these findings indicate that the plant extracts had beneficial effects without causing toxic effects on the liver or kidneys (Table 3).

**TABLE 3:** Biochemical parameters after 30 days *in vivo* trial of *O. compressa* extracts on different animal groups.

Biochemical parameters	Groups of rabbit					
	Normal (base line)	−Ve control (normal saline)	+Ve control (glibenclamide)	Oc.Aqu	Oc.MetOH	Oc.EtAc
Total Cholesterol	50.66 ± 1.21	287.7 ± 0.98	133.33 ± 2.19	233.45 ± 1.87	156.78 ± 3.17	133.34 ± 4.18
TAG	62.22 ± 2.18	179.3 ± 1.98	124.5 ± 1.89	116.2 ± 2.11	108.9 ± 1.87	134.3 ± 0.99
Bilirubin (mg/dL)	0.42 ± 0.83	1.5 ± 0.29	1.47 ± 0.37	1.56 ± 0.17	1.62 ± 0.36	1.61 ± 0.18
ALP (U/L)	17.7 ± 0.91	38.56 ± 0.88	37.33 ± 0.71	39.88 ± 0.59	43.33 ± 1.87	36.66 ± 2.19
ALT (U/L)	6.89 ± 0.39	14.33 ± 1.69	13.33 ± 1.59	15.33 ± 4.96	14.11 ± 4.87	15.1 ± 2.67
Phospholipids	7.87 ± 1.19	11.22 ± 0.75	16.7 ± 2.06	14.5 ± 2.68	15.5 ± 1.63	17.7 ± 1.78
Urea	83.49 ± 2.11	100.1 ± 1.28	98.8 ± 0.78	98.1 ± 1.79	105.2 ± 0.67	99.66 ± 0.38
Total proteins	8.54 ± 1.89	4.33 ± 0.78	6.44 ± 0.28	4.22 ± 0.33	3.98 ± 1.21	4.78 ± 1.19
Creatinine (mg/dL)	1.44 ± 1.28	2.33 ± 0.87	2.28 ± 0.19	2.46 ± 0.98	2.39 ± 0.38	2.27 ± 0.39
HDL (mg/dL)	54.47 ± 3.18	33.32 ± 3.48	38.87 ± 3.93	31.12 ± 3.59	33.34 ± 2.95	35.54 ± 1.97
LDL (mg/dL)	23.43 ± 1.18	33.34 ± 1.78	26.98 ± 1.78	28.88 ± 1.69	25.54 ± 2.17	25.66 ± 1.47
VLDL (mg/dL)	66.65 ± 2.18	139.98 ± 6.89	103.33 ± 4.56	122.2 ± 6.45	156.65 ± 7.87	201.1 ± 8.39
SGOT	59.93 ± 7.28	113.3 ± 3.31	116.5 ± 4.29	118.8 ± 9.87	121.4 ± 7.89	119.34 ± 6.39

Normal base line group: animals having no disease induction and no treatment; −ve control group: alloxan induced animals, treated with normal saline; = ve control group: alloxan induced animals, treated with Glibenclamide; Oc.Aqu group: alloxan induced animals treated with aqueous extract of *Octhochloa compressa* (500 mg/kg BW); Oc.MetOH; alloxan induced animals treated with methanol extract of *Octhochloa compressa* (500 mg/kg BW); Oc.EtAc: alloxan induced animals treated with ethyl acetate extract of *Octhochloa compressa*. (500 mg/kg BW).

### 3.4 Histopathological parameters

**FIGURE 5:**
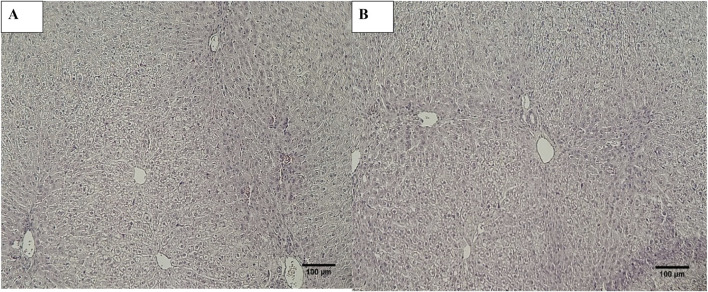
Histopathology of Liver. **(A)** Liver of diabetic animals treated with ethyl acetate extract of *O. compressa* (500 mg/kg BW) showing mild sinusoidal dilations and miild fatty change. **(B)** Liver of healthy animal with normal histopathology.

**FIGURE 6:**
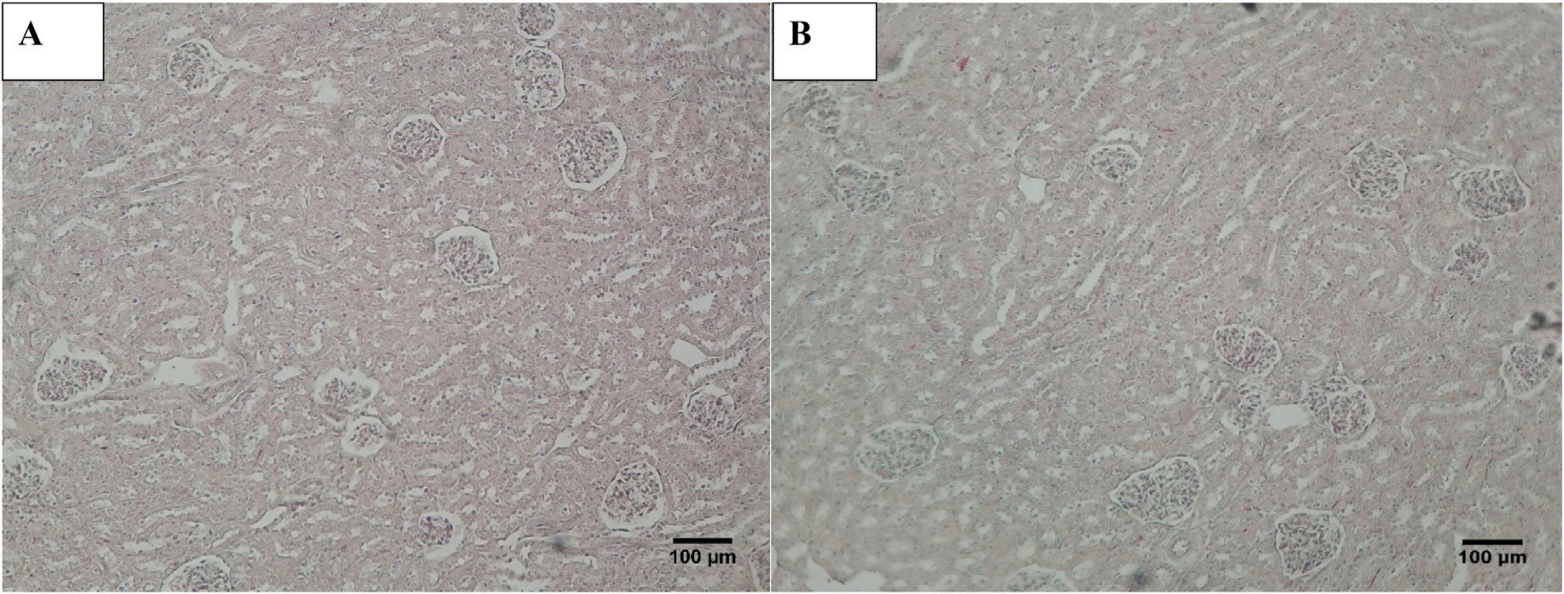
Histopathology of Kidney. **(A)** Kidney of diabetic animal treated with EtAc extract of *O. compressa* (500 mg/kg BW) showing mild tubular atrophy and congestion of capillaries. **(B)** KIdney of normal animal showing normal histology.

**FIGURE 7:**
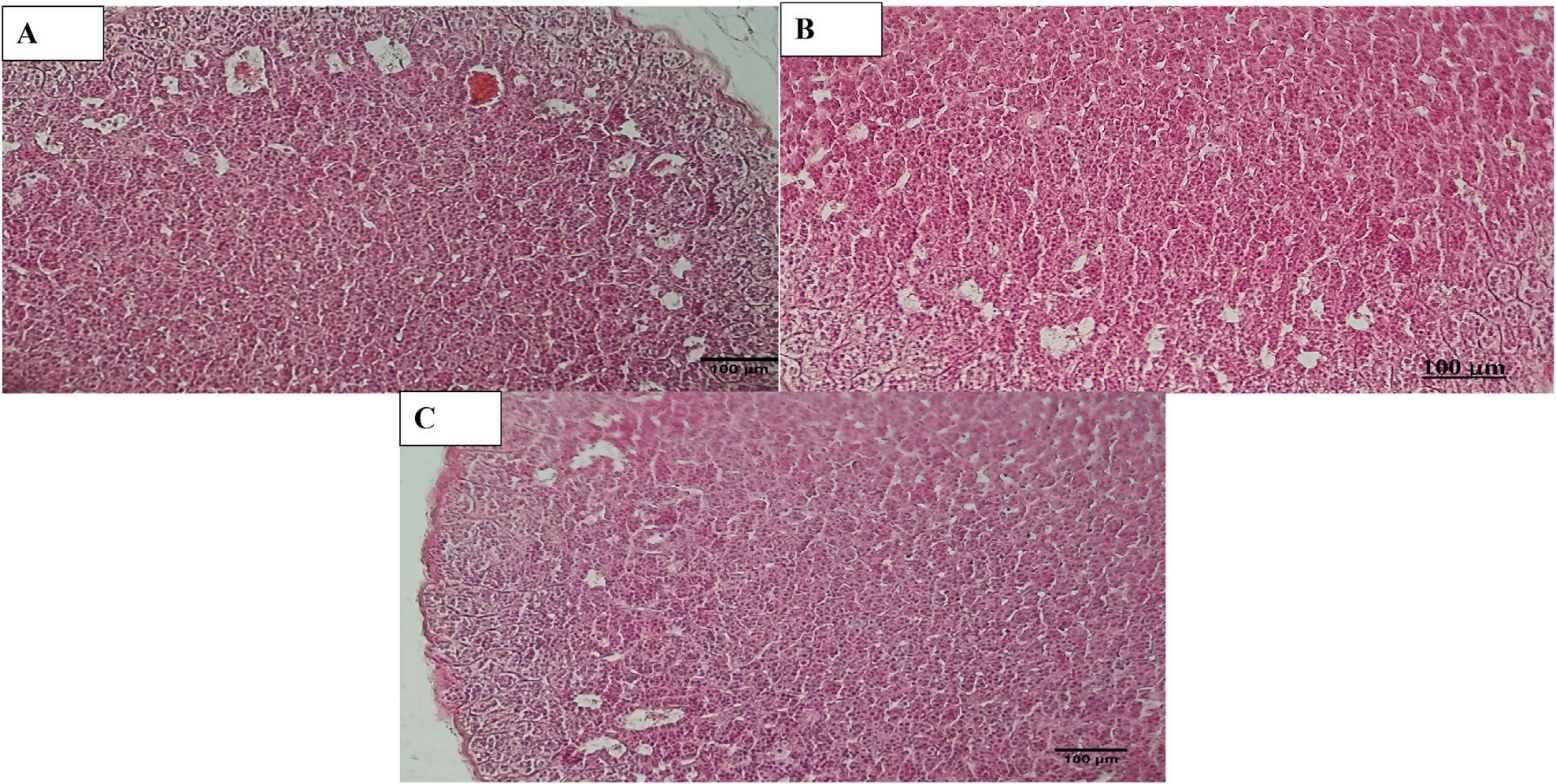
Histopathology of Adrenal. **(A)** Adrenal diabetic animal treated with EtAc extract of *O. compressa* (500 mg/kg BW) showing distortion in Zona glomerulosa, zona fasciculata and dilation in sinusoids in cortex. **(B)** Adrenal of diabetic animal treated with standard drug mild distortion in zona glomerulosa, zona fasciculata and dilation in sinusoids in cortex. **(C)** Adrenal of normal animal showing normal histopathology.

Histopathological examination revealed that in normal animals, the liver displayed typical characteristics, whereas diabetic animals exhibited mild liver changes such as fatty alterations and sinusoidal dilations in the cortex zone. The animals treated with *O. compressa* extracts displayed non-toxic effect on liver (Figure 5). Kidney histopathology in control animals showed normal structure, while diabetic animals treated with *O. compressa* extracts had minor tubular atrophy, mild glomerular sclerosis, and capillary congestion (Figure 6). Adrenal gland histopathology in normal animals appeared normal, but diabetic animals treated with *O. compressa* extracts displayed slight distortions in the cortex zones and sinusoidal dilation (Figure 7). These changes were likely attributed to the toxic effects of alloxan and diabetes, with no observed toxicity from the plant extracts themselves on the liver, indicating their safety.

### 3.5 HPLC polyphenol quantifications

**FIGURE 8:**
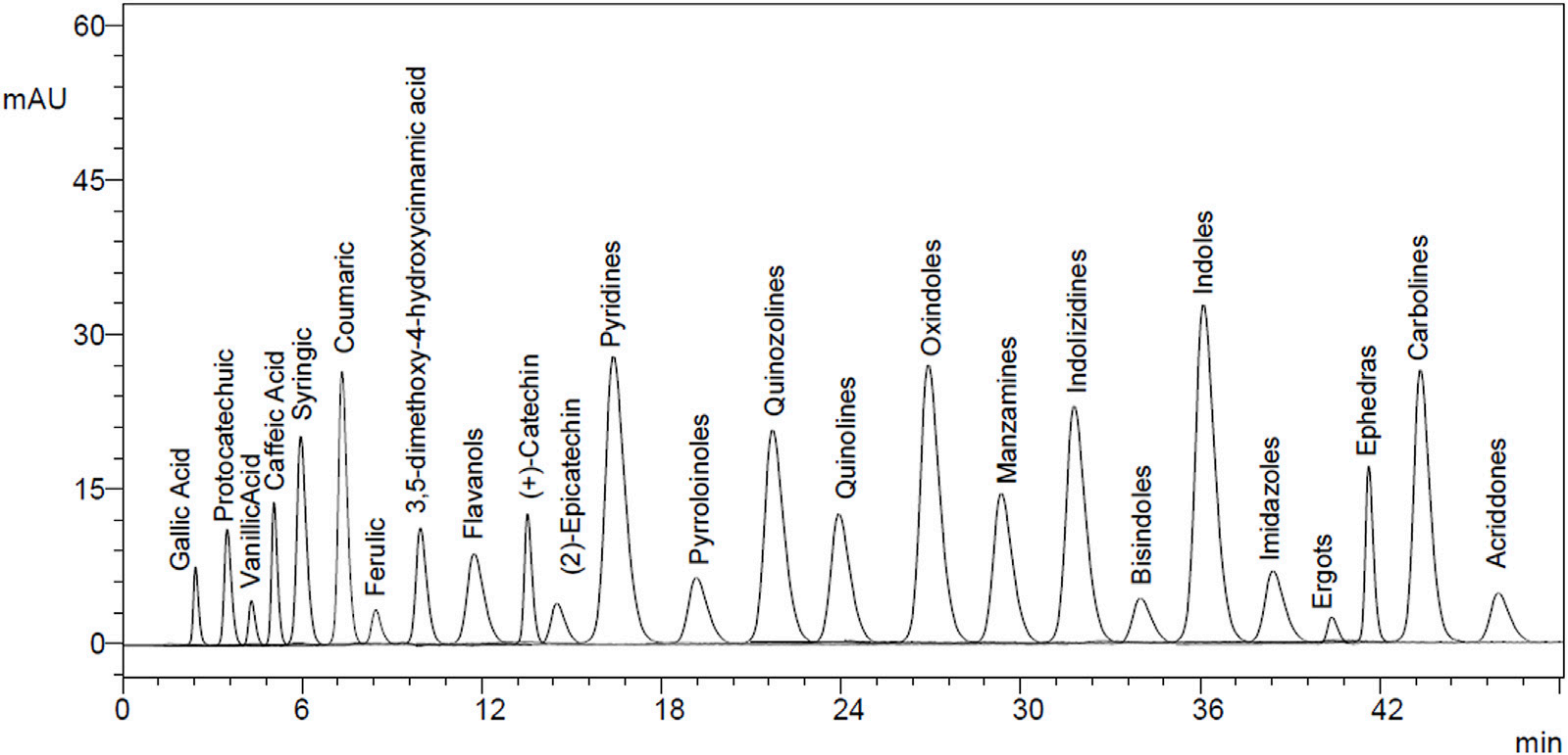
HPLC chromatogram of standard compounds.

**FIGURE 9:**
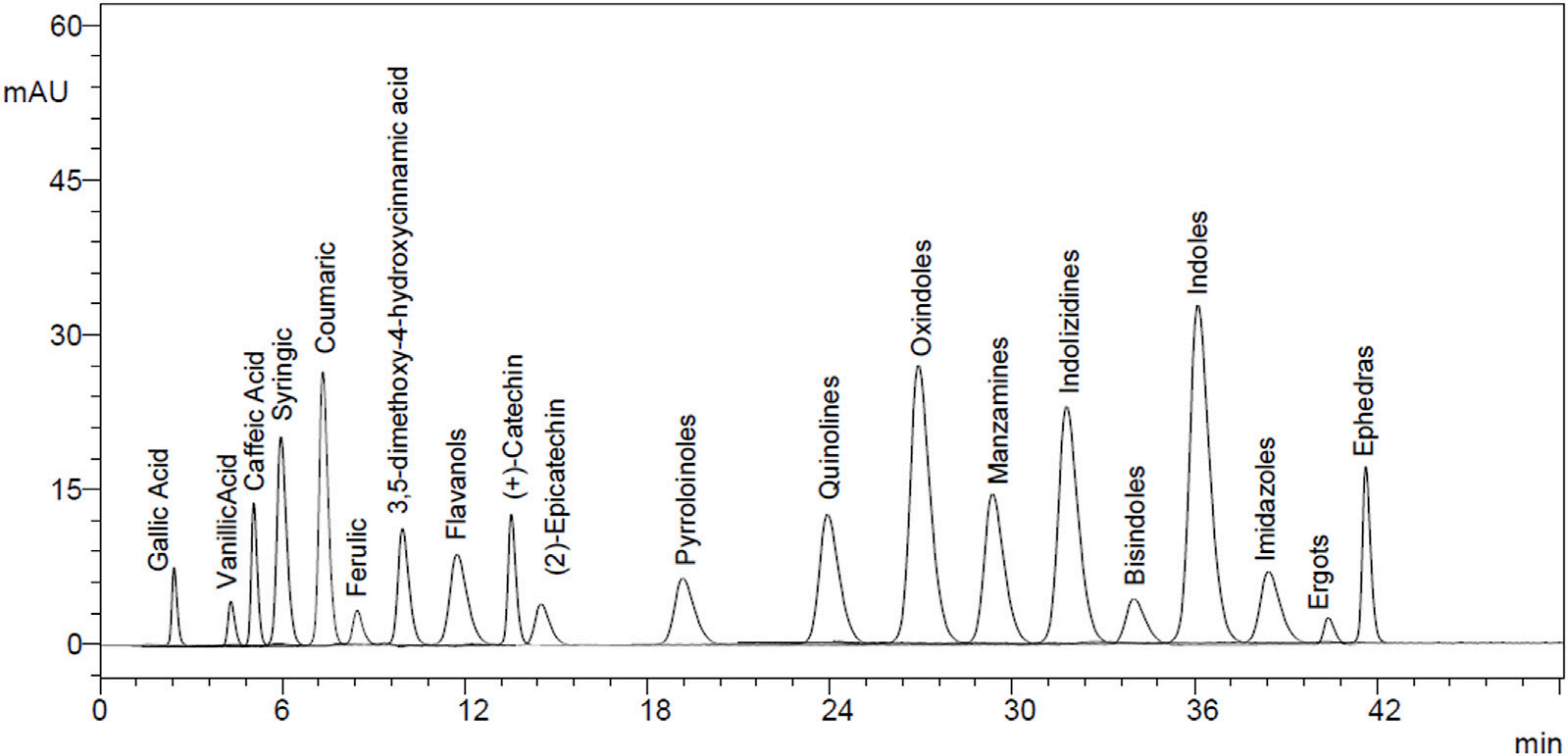
HPLC chromatogram of EtAc extract.

The EtAc extract of *O. compressa* exhibited the highest anti-diabetic potential was subjected to HPLC analysis to quantify polyphenols. The rationale for choosing these particular polyphenols—such as gallic acid, lignans, apigenin, tannins, and xanthones—was based on their well-documented pharmacological relevance, especially their antioxidant, anti-inflammatory, and anti-diabetic properties, as reported in the broader literature on medicinal plants. Although comprehensive phytochemical data for *O. compressa* are limited, our previous study (Aslam et al., 2021) provided preliminary profiling that informed the selection of these compounds for targeted quantification. This analysis revealed the presence of several compounds, including tannins (18.63 μg/mg), gallic acid (14.51 μg/mg), coumarin (7.16 μg/mg), chromone (10.95 μg/mg), xanthone (16.63 μg/mg), lignan (1.08 μg/mg), acridone (4.79 μg/mg), caffeine (4.41 μg/mg), carboline (10.01 μg/mg), ergots (4.29 μg/mg), imidazole (11.75 μg/mg), oxindole (8.59 μg/mg), quinoline (1.12 μg/mg), and apigenin (5.17 μg/mg) (Table 4; Figures 8, 9).

**TABLE 4:** HPLC quantifications of *O. compressa* EtAc extracts.

Ser. No	RT (min)	Peak area	Molecular formula	Molecular weight	Identified compound	Amount in (μg/mg)
1	1.252	1883950	C_72_H_52_O_46_	1701.2	Tannins	18.63
2	1.830	6841717	C_7_H_6_O_5_	170.12	Gallic Acid	7.51
3	2.090	3174978	C_9_H_6_O_2_	146.14	Coumarin	7.16
4	2.647	523433	C_9_H_6_O_2_	146.14	Chromone	10.95
5	2.883	879268	C_13_H_8_O_2_	196.19	Xanthone	16.63
6	3.247	482403	C_25_H_30_O_8_	458.5	Lignan	1.08
7	3.465	330087	C_13_H_9_NO	195.22	Acridone	4.79
8	3.759	257312	C_8_H_10_N_4_O_2_	194.19	Caffeine	4.41
9	3.984	421863	C_11_H_8_N_2_	168.2	Carboline	10.01
10	4.474	291277	C_23_H_27_N_3_O_6_	441.5	Ergots	4.29
11	5.008	551376	C_3_H_2_N_2_	68.077	Imidazole	11.75
12	7.788	149631	C_8_H_7_NO	133.150	Oxindole	8.59
13	8.252	102890	C_9_H_7_N	129.16	Quinoline	
14	9.665	50342	C_9_H1_7_N	139.24	Quinolizidine	1.12
15	11.407	53868	C_15_H_10_O_5_	270.24	Apigenin	5.17

### 3.6 *In silico* anti-diabetic study of *O. compressa* extract

**FIGURE 10:**
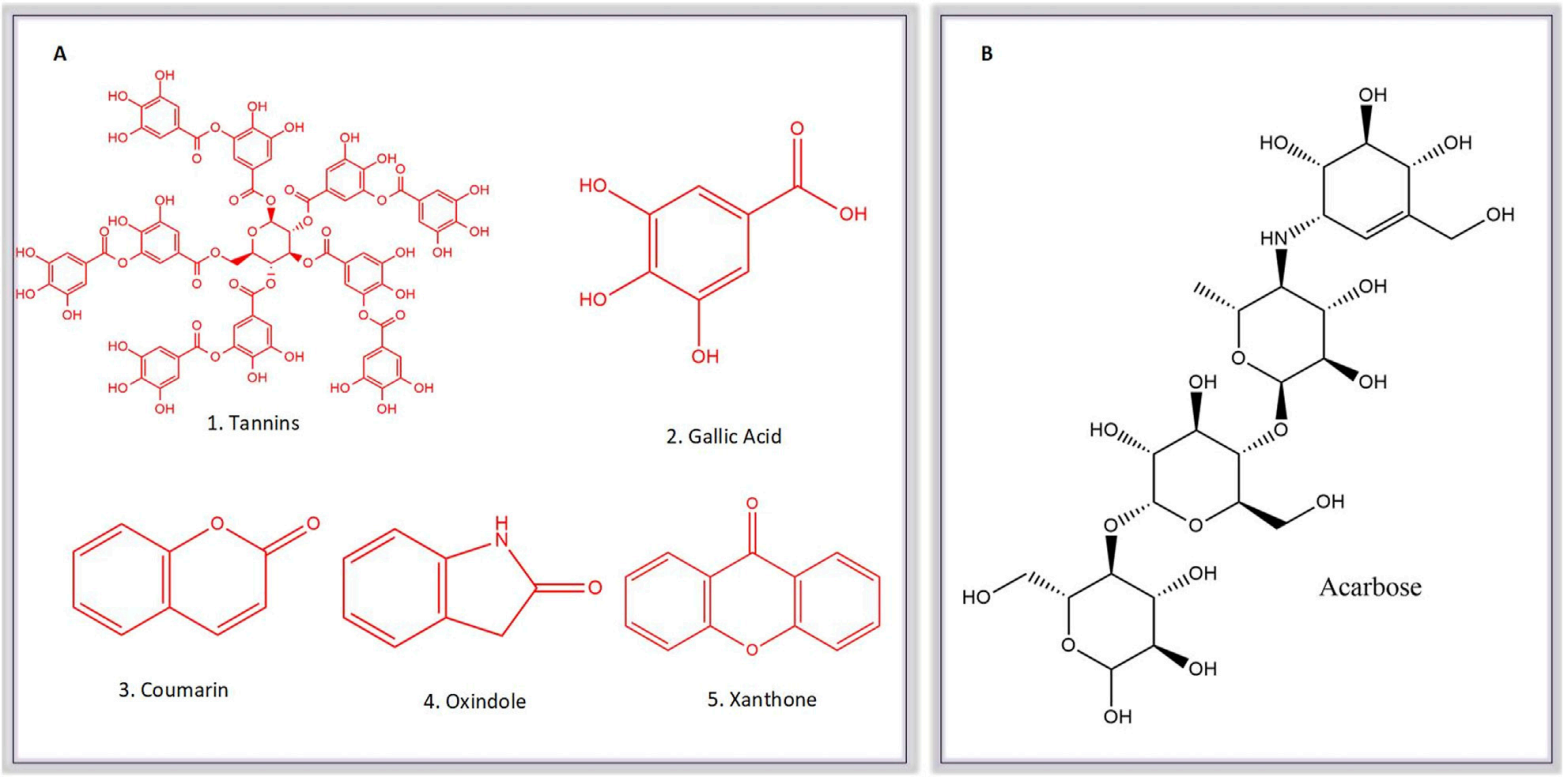
Molecular Structure of known Comounds (Tannins, Gallic acid, Coumarin, Oxindole and Xanthone) within the active sub-extracts **(A)** and acarbose **(B)**.

**FIGURE 11:**
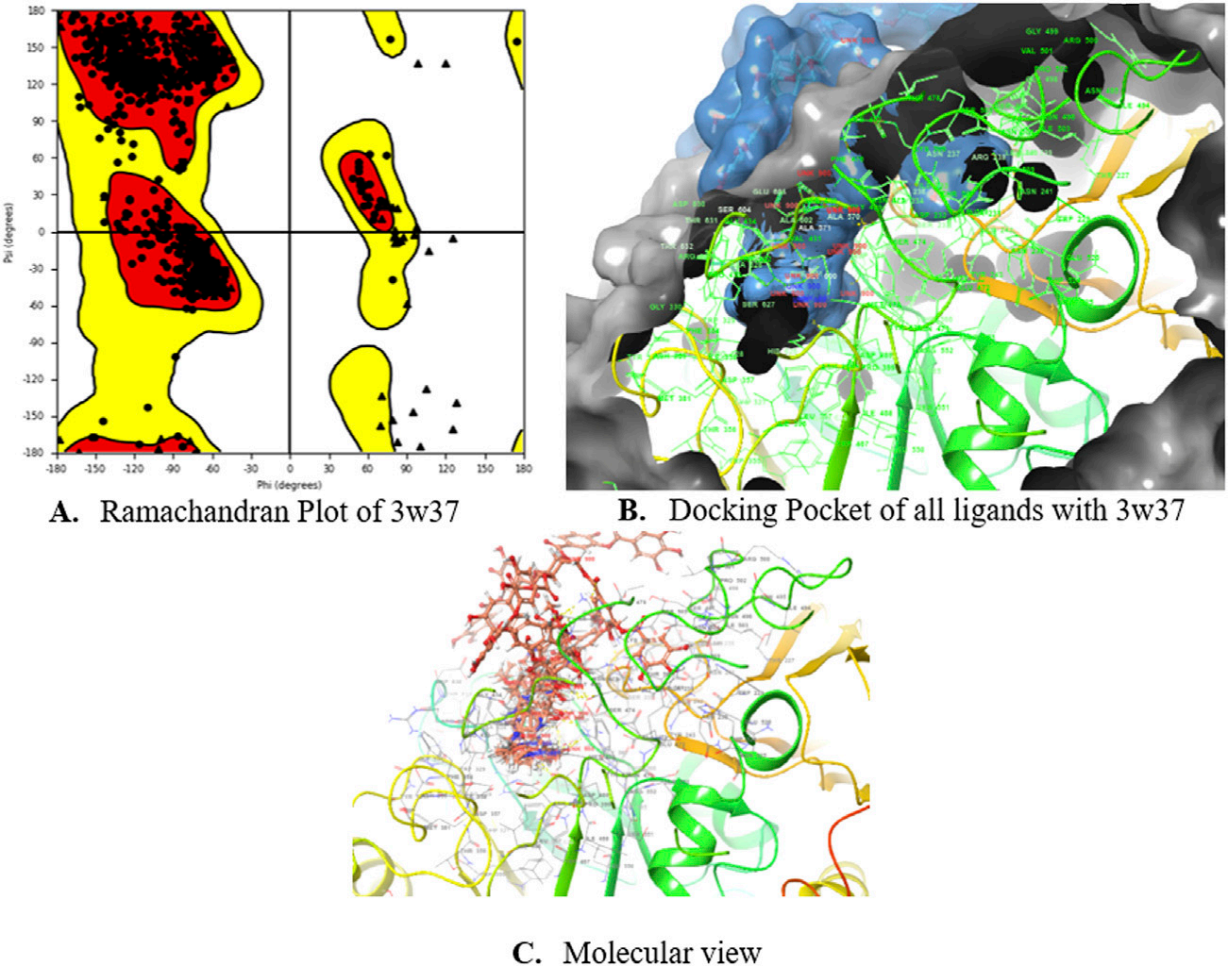
Ramachandran Plot **(A)**, Docking Pockets **(B)** and Molecular view **(C)** of glucosidase enzyme.

**FIGURE 12:**
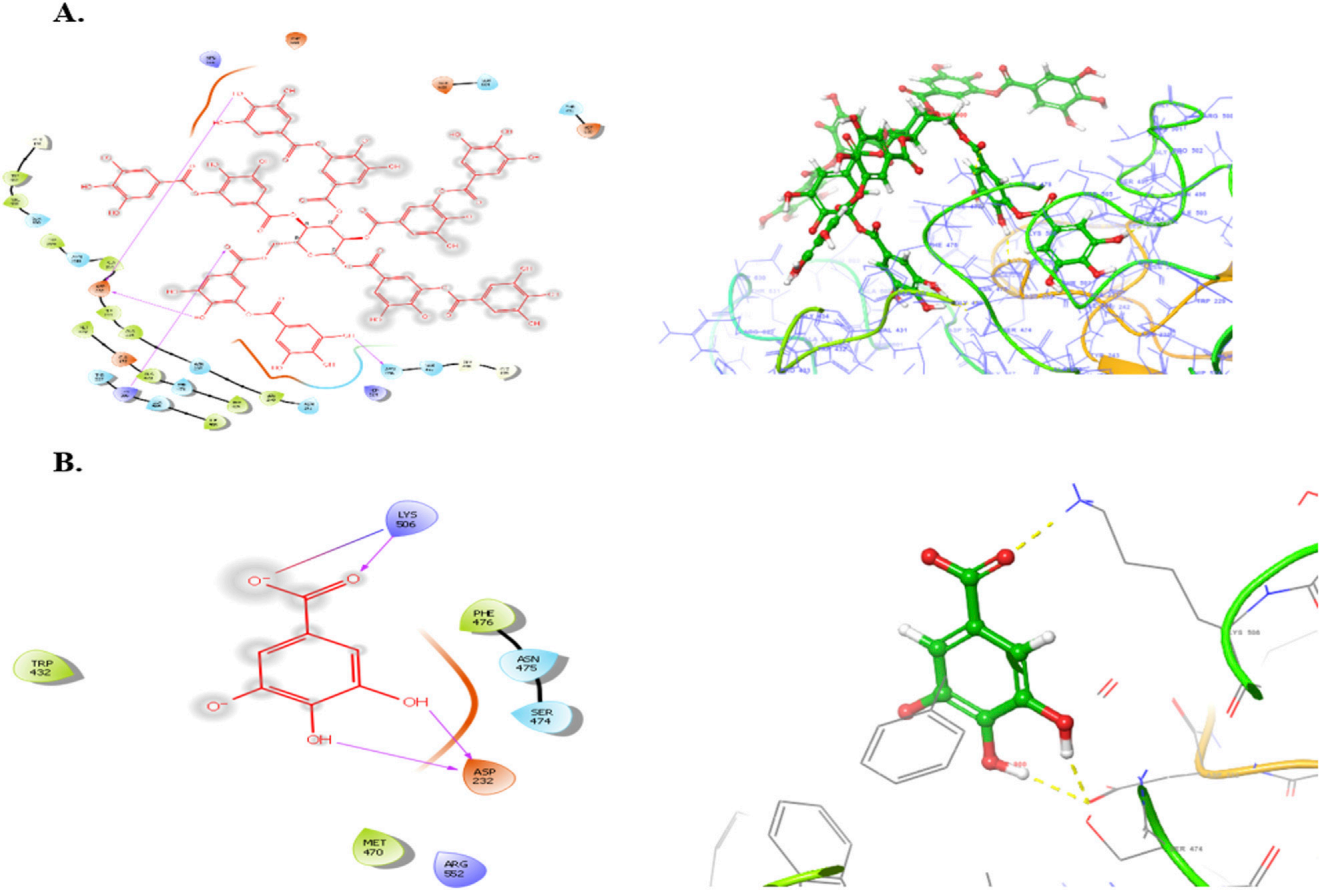
Binding poses o Tannins **(A)**, Gallic acid **(B)**, Coumarin with the active site reidues of α-glucosidase enzyme (3W37).

Subsequently, an attempt was made to conduct docking studies against the target protein (PDB ID: 3W37) using all identified compounds from the EtAc extract of *O. compressa;* including tannins, gallic acid, coumarin, oxindole, xanthone, apigenin, quinoline, chromone, quinolizidine, acridone, caffeine, carboline, imidazole and lignan (Table 4; Figure 10). Notably, 96.5% of the residues in the Ramachandran plots of *α-*glucosidase occupied preferred regions, confirming the accuracy of phi (φ) and psi (ψ) angles in the target protein’s coordinates (Figure 11A). The goal was to find optimal orientations and conformations that could interact effectively with the target protein (PDB ID: 3W37). Our assessment focused on binding interactions and binding energy values (Figures 11B,C). Among these compounds, tannins, gallic Acid, coumarin, oxindole, and xanthone (Figure 10) formed stable complexes with the target protein (3W37) with docking scores of −7.907, −6.59, −6.338, −6.328, and −6.07 kcal/mol, respectively (Table 5). A comparative analysis of structure-activity relationships (SAR) provided essential insights. For instance, tannins exhibited stable hydrogen bonding interactions with specific residues, including Asp232 and ASN496 within the active site of the target enzyme (3W37) (Figure 12A). Similarly, gallic acid showed stable hydrogen bonding interactions involving the dihydroxy groups with Asp232 and Lys506 (Figure 12B). Coumarin displayed significant hydrophobic interactions with specific residues, such as Trp432, Trp329, Met470, Asp469, Trp467, Asp568, Gly567, Trp565, Asp357, and Ile358. Additionally, oxindole demonstrated substantial hydrophobic interactions and stable hydrogen bonding interactions with Arg552, Met470, Asp469, Trp467, Trp565, Asp568, Asp357, ILE38, and Trp432. In the case of xanthone, pi-pi interactions with Trp432, Trp329, and Phe601, along with H-bonding with Arg552, were observed. In summary, our findings consistently ranked the stability of docked complexes in the following order: tannins < gallic acid < coumarin < oxindole < xanthone. Notably, the presence of dihydroxy and nitro groups emerged as key factors contributing to stable interactions within the binding pocket of the α-glucosidase enzyme.

**TABLE 5:** Docking score of ligands with protein 3w37.

Sr no	Pub Chem_ID	Entry name	Docking score	Energy	Amino acid interaction
1	16129778	Tannins	−7.91	−103.148	Asp232 and ASN496
2	370	Gallic Acid	−6.59	−84.50	Asp232 and Lys506
3	323	Coumarin	−6.34	−76.45	Trp432, Trp329, Met470, Asp469, Trp467, Asp568, Gly567, Trp565, Asp357, and Ile358
4	321710	Oxindole	−6.33	−74.20	Arg552, Met470, Asp469, Trp467, Trp565, Asp568, Asp357, ILE38, and Trp432
5	7020	Xanthone	−6.08	−68.98	Trp432, Trp329, and Phe601
6	5280443	Apigenin	−5.68	−62.97	-
7	7047	Quinoline	−5.61	−59.34	-
8	10286	Chromone	−5.57	−56.78	-
9	119036	Quinolizidine	−5.44	−52.19	-
10	2015	Acridone	−5.31	−49.37	-
11	2519	Caffeine	−5.14	−47.98	-
12	105078	Carboline	−5.13	−37.08	-
13	795	Imidazole	−5.11	−36.90	-
14	261166	Lignan	−4.03	−31.17	-

## 4 Discussions

Diabetes mellitus is a global public health concern with severe complications, including nephropathy, neuropathy, cardiovascular issues, and more, caused by either insufficient insulin production (Type 1) due to an autoimmune response or insulin resistance (Type 2), leading to elevated blood glucose levels and oxidative stress, emphasizing the importance of glucose control to prevent complications (Chen et al., 2019; Shi et al., 2019). Type-2 diabetes is expected to affect an additional 55.5 million people in Asia by 2023, particularly among middle-aged individuals (Gandhi et al., 2020). Recent studies emphasize the use of medicinal plants as a low-side-effect therapeutic addition in treating diabetes mellitus, particularly highlighting the effectiveness of phytochemicals like flavonoids in inhibiting α-glucosidase (Jugran et al., 2021; Patil et al., 2020). Traditional medicine, with a history spanning thousands of years, has been the primary healthcare system globally, especially in developing nations, where approximately 80% of people rely on medicinal plants for healthcare due to their accessibility, affordability, and effectiveness. Medicinal plants, containing bioactive phytochemicals with specific therapeutic properties, have gained popularity in treating various diseases (Anwar et al., 2021). Pakistan, with its diverse climates and abundant forests, supports nearly 6,000 plant species, with 12.5% recognized for their medicinal benefits, a percentage steadily rising due to local researchers’ interest in natural remedies (Tariq et al., 2020).

*α-*glucosidase, located in the small intestine’s mucosal epithelium, plays a key role in converting complex sugars into glucose, elevating blood glucose levels. Inhibiting α-glucosidase is effective in lowering blood glucose and mitigating post-meal hyperglycemia, making it a potential therapeutic approach for managing diabetes-related conditions such as hyperlipoproteinemia and obesity. Acarbose, a widely used *α*-glucosidase inhibitor, reduces blood glucose levels by limiting starch digestion in the intestine (Nazir et al., 2021; Shah et al., 2021). The data of *α*-glucosidase inhibition from *O. compressa* is not given in literature. In this study, six different extracts of *O. compressa* (Aqu, MetOH, *n*-But, EtAc, *n-*Hex and DCM) were tested for α-glucosidase inhibition at different concentrations. Among six different extracts, the EtAc and MetOH extracts of *O. compressa* exhibited potent inhibition of α-glucosidase with IC_50_ values of 190.6 ± 1.19 μg/mL and 281.0 ± 0.98 μg/mL, respectively, followed by Aqu extract with an IC_50_ of 380.5 ± 1.12 μg/mL. Number of phenolic compounds in plant extracts have been reported as α-glucosidase inhibitors, reduce glucose absorption in the intestine, and activate anti-hypoglycemic agents, aiding in blood glucose control and obesity management in Type-2 diabetes. Plant extracts also enhance glucose transport via GLUT4, crucial for maintaining glucose homeostasis in adipose tissue and skeletal muscles, offering potential anti-diabetic benefits (Riyaphan et al., 2021).

The poor control of glucose is a key factor of diabetes mellitus, can result in severe long-term complications if not managed effectively. The pancreas functions as a “sensing organ” that monitors blood glucose levels and releases insulin when necessary to regulate energy and nutrition. Various factors can disrupt this crucial mechanism (Hussain et al., 2020). In the study, alloxan-induced diabetes was employed as a model, leading to pancreatic inflammation and the degeneration of Langerhans islet beta cells, ultimately causing hyperglycemia, reduced insulin production, and increased lipid levels. Previous research on alloxan-induced diabetic mice yielded similar outcomes (Maqbool et al., 2021). Presently, various oral antidiabetic medications, such as insulin, sulphonylureas, metformin, α-glucosidase inhibitors, and troglitazone, are available for diabetes treatment. Plant-based treatments offer an alternative approach with potential applications in various domain (Khan et al., 2022). *O. compressa* has previously demonstrated remarkable anti-inflammatory activity both *in vitro* and *in vivo*, along with a low toxicity profile (Aslam et al., 2021). The hypoglycemic effects of *O. compressa* crude extracts were evaluated on healthy and alloxan-induced diabetic rabbits. Results showed no change in fasting blood glucose levels in the normal base line group, while the negative control group exhibited prolonged hyperglycemia. The standard group treated with glibenclamide showed significant reductions in blood glucose levels. The alloxan induced animal treated with Aqu, MetOH and EtAc extracts of *O. compressa* at the doses of 250 mg/kg BW, 500 mg/kg BW and 750 mg/kg BW. Among these extracts, the 750 mg/kg BW of Aqu extract had a slight effect, while the 500 mg/kg BW of MetOH and EtAc extracts demonstrated significant hypoglycemic effects, with the 500 mg/kg BW of EtAc extract being the most effective in maintaining blood glucose levels over the 30-day trial in diabetic-induced rabbits. These results align with a previous study that observed significant hypoglycemic effects in alloxan-induced hyperglycemic rabbits upon administering the ethyl acetate extract of *Ruellia tuberosa* (Ullah et al., 2012). Likewise, the ethyl acetate extract of *Caralluma tuberculata* demonstrated noteworthy hypoglycemic effects in alloxan-induced hyperglycemic rabbits (Sultan et al., 2014). This effect of EtAc extract is attributed to its rich content of bioactive compounds which was identified by HPLC analysis, including tannins, gallic acid, coumarin, chromone, xanthone, lignan, acridone, caffeine, carboline, ergots, imidazole, oxindole, quinoline, and apigenin. The presented findings are consistent with previous studies. For instance, gallic acid and catechin have been identified in the EtAc extract of Rubus ulmifolius leaves, demonstrating significant antioxidant and antibacterial activities, which are beneficial in managing diabetes-related oxidative stress and infections (Bramki et al., 2024). Additionally, the EtAc fraction of *Ficus lutea* leaf extract, rich in polyphenols, exhibited potent α-glucosidase inhibitory activity and enhanced insulin secretion, highlighting the therapeutic potential of such bioactive compounds in diabetes management (Olaokun et al., 2016). Gallic acid, tannins, and apigenin have been identified as compounds that stimulate insulin release from pancreatic cells and inhibit α-glucosidase, potentially elucidating the antidiabetic mechanism of *O. compressa* extracts. A prior investigation on *N. procumbens* by our recent study also reported comparable polyphenolic content, supporting these findings (Aslam et al., 2023a). These compounds utilize various mechanisms, including suppressing liver gluconeogenesis, enhancing glycolysis, inhibiting intestinal glucose absorption, promoting insulin release, and inhibiting α-glucosidase enzymes (Riyaphan et al., 2021; Shi et al., 2019). Natural products with phenolic compounds have been explored for their ability to inhibit α-glucosidase enzymes, activate anti-hypoglycemic agents, and combat hyperglycemia and obesity. Managing type-2 diabetes involves incorporating low glycemic index foods and dietary fibers, with plant extracts contributing to glucose regulation via GLUT4 protein. Given the increasing prevalence of type-2 diabetes, especially in Asia, utilizing plant ingredients with specific phytochemical profiles in supplementary foods offers an economical approach to management (Patil et al., 2020; Shanak et al., 2022; Tariq et al., 2020).

Exposure to foreign substances can harm cells and vital detoxifying organs, such as the kidney, liver, and pancreas, leading to biochemical substance release into the bloodstream (Manivannan and Shopna, 2017). Elevated blood creatinine levels are indicative of renal toxicity, suggesting impaired waste material removal from damaged kidneys (Elangovan et al., 2019). The study examined various biochemical factors in control, diabetic, and treated groups. It found that alloxan-induced diabetes increased bilirubin levels, but *O. compressa* extracts with anti-hyperbilirubinemia potential effectively reduced these levels. Importantly, the administration of plant extracts did not significantly impact serum creatinine levels, indicating no adverse effects on kidney function compared to the normal control group. The study’s findings indicated that diabetes induction had detrimental effects on kidney function, as evidenced by various biomarkers (Gupta et al., 2020). Although elevated liver biomarkers such as AST and ALT typically suggest liver toxicity, in this case, diabetes induction and subsequent treatment with plant extracts did not negatively impact the liver (Mechchate et al., 2021). The observed variations in these markers remained within the normal physiological range and held no clinical significance for the species. Multiple studies have established a connection between elevated blood sugar levels in diabetes and increased blood lipid levels, known as hyperlipidemia (Karigidi and Olaiya, 2020; Rodrigues et al., 2020). Elevated cholesterol levels are a major risk factor for coronary heart disease and a leading contributor to diabetic complications. Untreated diabetic groups often exhibit higher levels of triglycerides and cholesterol, indicative of significant alterations in lipid metabolism and structure. While diabetes disrupts cholesterol cellular metabolism, the exact mechanisms remain unclear. The substantial rise in triglycerides in diabetic mice is likely a consequence of reduced plasma insulin levels, which typically stimulate lipoprotein lipase and triglyceride breakdown. Given the association between diabetes and elevated triglycerides, various plant species have been tested and proven effective in lowering triglyceride levels (Mechchate et al., 2020; Vieira et al., 2020). The treatment with *O. compressa* led to a notable reduction in triglyceride and cholesterol levels in the treated animals, indicating potential benefits for both blood sugar and lipid regulation.

Oral plant extract administration to alloxan-induced diabetic rabbits resulted in minor histopathological changes compared to normal controls. Normal liver tissue displayed typical hepatic cells, while diabetic animals maintained lobular architecture but exhibited mild alterations, including fatty changes and sinusoidal dilations. In the kidneys, control animals had a normal structure, but diabetic animals treated with plant extracts showed mild changes like minor tubular atrophy, slight glomerular sclerosis, and capillary congestion. Additionally, the adrenal glands in normal animals appeared normal, while those in diabetic animals treated with *O. compressa* extracts displayed mild distortions in the zona glomerulosa and zona fasciculata, along with sinusoidal dilation in the cortex. These morphological changes were primarily attributed to the toxic effects of alloxan and diabetes (Khalaf et al., 2021). Notably, the study found that the doses of *O. compressa* used were non-toxic and did not harm the liver. These mild changes in the kidney and adrenal glands were likely due to alloxan’s impact on diabetic-induced rabbits and align with another study that demonstrated the non-toxic effects of the pomegranate seed oil through histopathological investigations (Ibrahim et al., 2021). Consequently, it can be concluded that the extracts are non-toxic and have the potential to mitigate the maximal toxic effects of alloxan in diabetic-induced rabbits.

Computational approaches such as molecular docking are crucial in modern drug discovery, especially for targets like α-glucosidase, an enzyme central to carbohydrate metabolism and postprandial blood glucose regulation (Vijayakumar et al., 2023). In this *in silico* study, several compounds identified via HPLC from the EtAc extract of *O. compressa*—notably tannins, gallic acid, coumarin, oxindole, and xanthone—demonstrated strong binding affinity with α-glucosidase (PDB ID: 3W37), with docking scores of −7.907, −6.59, −6.338, −6.328, and −6.07 kcal/mol, respectively. These values indicate the potential for effective enzyme inhibition, comparable in some cases to the clinically used α-glucosidase inhibitor acarbose, which typically scores between −8.5 and −9.2 kcal/mol (Nada et al., 2024). Tannins, the top-scoring compounds, showed extensive hydrogen bonding with key catalytic residues such as Asp232 and Asn496. These interactions are attributed to their multiple phenolic groups, which facilitate anchoring within the enzyme’s active site—mirroring the mechanism of tannic acid observed in prior studies (Agrawal et al., 2022). Similarly, the gallic acid formed stable hydrogen bonds with Asp232 and Lys506 through its dihydroxy groups, underscoring the importance of hydroxyl functionality in molecular recognition. These interactions correlate with *in vivo* findings showing that tannin- and gallic acid-rich extracts improve glucose homeostasis and insulin sensitivity in type 2 diabetes models (Huang et al., 2019; Shanak et al., 2022). Gallic acid diagnosed as significantly inhibitors of *α*-glucosidase and *α*-amylase (Palanuvej et al., 2009). Further, coumarin, oxindole, and xanthone (particularly mangiferin, a known xanthone derivative) displayed dominant hydrophobic and π–π stacking interactions with aromatic residues such as Trp432, Trp329, and Phe601. These interactions are well-documented in stabilizing enzyme-inhibitor complexes, and their presence here suggests a robust binding conformation. Oxindole also contributed additional hydrogen bonds via Arg552 and Met470, adding stability to the interaction. Importantly, compounds like coumarin and mangiferin have been shown to outperform acarbose in α-glucosidase inhibition and are capable of reducing postprandial glucose levels and improving lipid profiles in diabetic models (Tshiyoyo et al., 2022; Yan et al., 2014). Collectively, these results suggest that the antidiabetic activity of *O. compressa* extract may be attributed to competitive inhibition of α-glucosidase by its phytochemicals. The molecular interactions—particularly hydrogen bonding with catalytic residues and hydrophobic contacts with stabilizing residues—resemble those of known inhibitors and offer a mechanistic basis for delaying carbohydrate digestion and glucose absorption. Thus, these findings support the potential of *O. compressa* as a complementary therapeutic agent in managing Type 2 diabetes and associated metabolic disorders.

## 5 Conclusion

This study found that *O. compressa* exhibits promising anti-diabetic properties, validated through *in vitro, in silico* and *in vivo* experiments. Specifically, the ethyl acetate (EtAc) and methanol (MetOH) extracts of *O. compressa* displayed remarkable inhibition of α-glucosidase. Furthermore, *in vivo* studies on an alloxan-induced hyperglycemic rabbits, demonstrated that among all 3 tested extracts, the EtAc extract exhibited significant dose-dependent anti-diabetic effects, positively impacting the pancreas, liver, and kidney, as evident from the concentrations of relevant biochemical markers. *In silico* molecular docking revealed that the EtAc extract’s anti-hyperglycemic effects can be attributed to the presence of tannins, gallic acid, coumarin, oxindole, apigenin, and xanthone. These findings highlight the potential of *O. compressa* as a natural and promising solution for managing diabetes and its associated complications. Future research should focus on isolating and characterizing these bioactive compounds, elucidating their mechanisms of action, assessing long-term safety, and conducting clinical trials to fully explore the therapeutic potential of *Octhochloa compressa* in diabetes management.

## Data Availability

The original contributions presented in the study are included in the article/supplementary material, further inquiries can be directed to the corresponding authors.
